# Modulate the impact of the drowsiness on the resting state functional connectivity

**DOI:** 10.1038/s41598-024-59476-8

**Published:** 2024-04-15

**Authors:** Marc Joliot, Sandrine Cremona, Christophe Tzourio, Olivier Etard

**Affiliations:** 1 GIN, IMN UMR5293, CEA, CNRS, Université de Bordeaux, Bordeaux, France; 2 BPH, U1219, INSERM, Université de Bordeaux, Bordeaux, France; 3 Normandie Université, UNICAEN, INSERM, COMETE U1075, CYCERON, CHU Caen, 14000 Caen, France

**Keywords:** Resting-state, fMRI, Drowsiness, Functional connectivity, Neuroscience, Cognitive neuroscience

## Abstract

This research explores different methodologies to modulate the effects of drowsiness on functional connectivity (FC) during resting-state functional magnetic resonance imaging (RS-fMRI). The study utilized a cohort of students (MRi-Share) and classified individuals into drowsy, alert, and mixed/undetermined states based on observed respiratory oscillations. We analyzed the FC group difference between drowsy and alert individuals after five different processing methods: the reference method, two based on physiological and a global signal regression of the BOLD time series signal, and two based on Gaussian standardizations of the FC distribution. According to the reference method, drowsy individuals exhibit higher cortico-cortical FC than alert individuals. First, we demonstrated that each method reduced the differences between drowsy and alert states. The second result is that the global signal regression was quantitively the most effective, minimizing significant FC differences to only 3.3% of the total FCs. However, one should consider the risks of overcorrection often associated with this methodology. Therefore, choosing a less aggressive form of regression, such as the physiological method or Gaussian-based approaches, might be a more cautious approach. Third and last, using the Gaussian-based methods, cortico-subcortical and intra-default mode network (DMN) FCs were significantly greater in alert than drowsy subjects. These findings bear resemblance to the anticipated patterns during the onset of sleep, where the cortex isolates itself to assist in transitioning into deeper slow wave sleep phases, simultaneously disconnecting the DMN.

## Introduction

Functional imaging during resting state (RS) refers to a behavioral and physiological state experienced when deprived of meaningful cognitive stimulation^1–3^. Utilizing functional magnetic resonance imaging (fMRI), researchers have observed regional hemodynamic low-frequency oscillations (LFOs)^4^, which exhibit similarity between distant brain regions. Subsequent investigations have revealed that this signal extends throughout the cortical, subcortical, and cerebellar gray matter. Moreover, functional connectivity (FC, see review^5^), denoting the signal similarity between regions, has emerged as an indicator of the cognitive anatomo-functional organization of the brain^6^.

The RS involves at least two processes of particular interest to us, arising from past and present cognitive activities. The first probably involves a Hebbian mechanism due to a history of prior co-activations^7^. The second, also linked to synaptic activity, would be related to the ongoing cognition during fMRI acquisition. These two processes are not mutually exclusive, as demonstrated in earlier studies by Grecius et al.^8^. The pseudo-periodic nature of LFOs suggests a stronger association with the first process. Indeed, investigations have explored and observed modulations of FC with genetic/developmental factors (e.g., sex), learning/cognitive skills^9,10^ and neuropsychiatric brain diseases^11^. The second process is self-oriented activity and a mental state associated with mind-wandering^12^ that can be modulated or abolished by drowsiness or sleep. In imaging studies, the cognitive activity has been described as “random episodic silent thinking (REST)”^1^ or “conscious RS”^2,13^. Capturing this “random” process is challenging since it involves a continuous flow of thoughts without any external triggers. Nevertheless, certain studies have reported variability in FC associated with the content of these thoughts^14–18^. However, even when investigating life events or ongoing cognition, the linked variability in FC accounts for only a tiny portion of the observed variance^15^.

It is important to note that fMRI acquisitions are subject to other sources of variability, and determining which factors should be accounted for remains an ongoing topic of research and debate. In addition to the sources of FC variability mentioned above, four undesired sources exist instrumental artifacts, subject movement-related artifacts, non-specific tissue artifacts, and physiological artifacts. Each source requires specific correction methodologies, comprehensively reviewed by Caballero-Gaudes et al.^19^.

The database we study here comes from the MRi-Share cohort^20^ and consists of 1722 brain scans acquired during a 15-min functional magnetic resonance imaging (fMRI) session using a 3 Tesla scanner, with participants instructed to keep their eyes closed to increase the likelihood of entering a mind-wandering state. As Tagliazucchi et al.^21^ demonstrate, mind-wandering promotes sleep, and FCs were increased in the lightest none rapid eye movements (NREM) sleep stages compared to the awake state. Accordingly, through a post-acquisition retrospective questionnaire on their cognitive activities during the scan, some MRi-Share participants reported to have experienced periods of drowsiness during the fMRI acquisition^22^. Meanwhile, we noted significant residual variability in FC, even after applying the correction techniques mentioned earlier. Therefore, we hypothesize that drowsiness enhances both the FCs and their variability across the sample of subjects.

A literature review strengthens the hypothesis that a difference in vigilance could account for a significant part of the observed variability in FCs. Numerous changes have been observed when studying the transition from wakefulness to sleep using RS-fMRI. The global blood oxygen level-dependent (BOLD) signal, measured across the entire brain, tends to increase as NREM sleep deepens^23^, whereas global cerebral blood flow measured by other techniques, such as positron emission tomography, decreases concurrently^24^. These observations collectively suggest a neurovascular decoupling induced by NREM sleep. Although brain networks revealed by RS-fMRI analysis are present across all sleep stages^25^, the strength of connectivity within the networks decreases during the deepest NREM stage, and specific sub-networks are no longer observed^26^. However, this decrease is not a continuous phenomenon along the wake-sleep axis, as studies have shown an increase in global connectivity during the initial sleep phase^27,28^. Furthermore, the temporal dynamics of brain networks are altered by changes in alertness, with transitions between FC states occurring more frequently during wakefulness compared to the deepest NREM sleep stages^29,30^. The lightest NREM sleep stage is distinct as it does not correspond to any single state or group of states identified through dynamic analysis^30^. Last, the microsleep episodes have been recently thoroughly investigated by Soon et al.^31^ albeit in eyes open condition, and the authors concluded that it should be considered when interpreting the FC. In summary, studies investigating sleep using MRI techniques are limited due to their inherent complexity, but they consistently reveal changes in brain FC compared to wakefulness. These changes do not form a continuous spectrum across the wake-sleep axis, leading us to hypothesize that they can be observed as soon as the initial signs of drowsiness appear, a state that frequently occurs during an RS-fMRI scan^21^.

Our interest of both past and ongoing behavioral aspects^10^ led us to measure the effects of vigilance on FC and identify the best method(s) to modulate these effects.

First, we compared FC preprocessed using the state-of-the-art minimal methodology^19^ in individuals who experienced drowsiness and those who remained alert during the whole fMRI acquisition. To distinguish drowsy from alert participants within our extensive dataset, we took advantage of the knowledge that during sleep, not only the electrical activities of neurons change but also the autonomic regulation^32^. These latter modifications make it possible to use the respiratory signal alone to classify sleep stages^33,34^ even in complex environments such as an intensive care unit^35^. Falling asleep gives rise to specific respiratory disturbances, including reduced chemoreceptor sensitivity and delayed feedback loops. These disturbances result in respiratory control system instability and periodic breathing characterized by alternating short episodes of hyperventilation and hypoventilation^36^. Although the exact occurrence of these phenomena as individuals fall asleep is unknown, we leveraged the opportunity presented by our large dataset, all of which was accompanied by photoplethysmography (PPG^37^) and respiratory belt measurements, to categorize subjects into three groups: those experiencing drowsiness most of the time during fMRI, those remaining fully alert, and those with a mixed or undetermined state.

Second, we examined four methods that could modulate the vigilance-induced FC differences between the two sets’ data. We used two methods of time series corrections and two methods of FC corrections. The first correction method was based on the Physiological Response Function (PRF)^38^, including RETROICOR (retrospective correction)^39^. The rationale of this method is to remove all that is periodic at the frequency of the heart and the respiration (RETROICOR) and all hemodynamic response to their fluctuations (PRF). The second correction method is the widely used and controversial global signal regression (GSR, see Murphy et al.^40^ for a review). The methodology is simple and requires linear regression removal from each voxel time series of the BOLD time course of the brain. This correction can be very efficient in the context of a systemic event that could affect the whole brain. However, following the idea that some information related to the studied process may be present in the global signal and thus the data “over-cleaned’ by the previous method, we designed two methodologies that rely on a more parsimonious correction acting directly on the FC's values and not the time series.

## Methods

### Data acquisition

The MRI datasets used in this study were obtained from a subset of the i-Share (internet-based Student Health Research, www.i-share.fr) protocol, which aims to investigate the health of university students. Of the 30,000 enrolled students in i-Share, 1870 volunteered to participate in the MRI study, forming the MRi-Share cohort^20^. The MRi-Share study was conducted with the approval of the “Comité de Protection des Personnes Sud-Ouest et Outre-Mer III” on 24 August 2015 (CPP2015-A00850-49), informed consent was obtained from all the subjects and all methods were performed in accordance with the relevant guidelines and regulations**.** After excluding subjects who did not complete the scanning session and those with incidental findings, a final sample of 1722 subjects remained for further analysis.

From the three acquired modalities, namely anatomical, diffusion, and functional MRI, we extracted ten sequences for this study. The T1-weighted images were obtained using a sagittal 3D magnetization-prepared 180-degree radio-frequency pulses and rapid gradient-echo (MP RAGE) sequence with the following parameters: repetition time (TR)/echo time (TE)/inversion time (TI) = 2000/2.0/880 ms and a voxel size of 1mm^3^. For fMRI geometric distortion correction, we utilized eight B0 diffusion mapping sequences as described in Tsuchida et al.^20^. The RS-fMRI acquisition consisted of a 15-min scan with 1058 volumes using a 2D axial Echo Planar Imaging (EPI) sequence (TR/TE = 850/35.0 ms, Flip Angle = 56°, voxel size 2.4^3^ mm^3^). This acquisition was performed on a 3 T Siemens scanner with a 64-channel head coil, utilizing the “Minnesota” multiband sequence with six times acceleration.

Among the final selected sample of subjects, 1640 provided exploitable simultaneous recordings of two physiological signals along with their fMRI data: blood volume changes in the microvascular bed of finger tissue using PPG^37^ and breathing movements using a pneumatic abdominal respiration transducer. During the RS scan, participants were instructed to keep their eyes closed, relax, avoid movement, remain awake, and allow their thoughts to come and go freely. Compliance with these instructions was verified using a self-report questionnaire administered at the end of the RS scanning session^22^.

### Physiological signal processing and vigilance assessment

**Figure 1:**
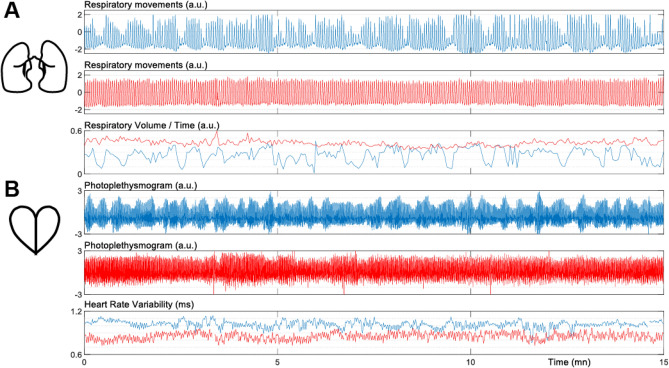
Respiratory (**A**) and heart (**B**) patterns of typical drowsy (blue) and alert (red) subjects (a.u.: arbritary unit).

The physiological signals of the 1640 subjects underwent initial visual screening to identify acquisition artifacts, this resulted in the exclusion of 270 (16%) subjects from further analysis. The second visual analysis focused on the respiratory signal to identify a characteristic pattern of drowsiness, characterized by alternating waxing and waning in the amplitude of abdominal respiratory movements. Previous research by Trinder et al.^41^ demonstrated that these amplitude alternations are associated with transitions between wakefulness and lightest NREM sleep stage, with decreases in ventilation during wakefulness to N1 state transitions and increases during N1 to wake state transitions. Therefore, for the group of subjects exhibiting oscillatory breathing indicative of drowsiness, their respiratory signal envelopes had to show clear modulation of more than 50% of the amplitude with a period of approximately 30 s^36^ over more than half of the resting test duration as illustrated with top plot of Fig. 1A. In contrast, for the group of alert subjects, the respiratory signals were required to exhibit minimal envelope modulation, as shown in the middle plot of Fig. 1A. The opposition of these two patterns is highlighted on the third plot, representing the estimated ventilatory flow of these two subjects. In the drowsy subject, the flow oscillates with a period of around 30 s, whereas in the alert subject, the estimated ventilatory flow is globally stable. Based on these criteria, we identified 164 participants out of the initial 1370 as either showing patterns of drowsiness (N = 68, 40W/28 M, age 21.9 ± 2.6 mean ± sd) or alertness (N = 96, 80W/16 M, age 22.1 ± 2.5 mean ± sd) based on their respiratory patterns.

**Figure 2:**
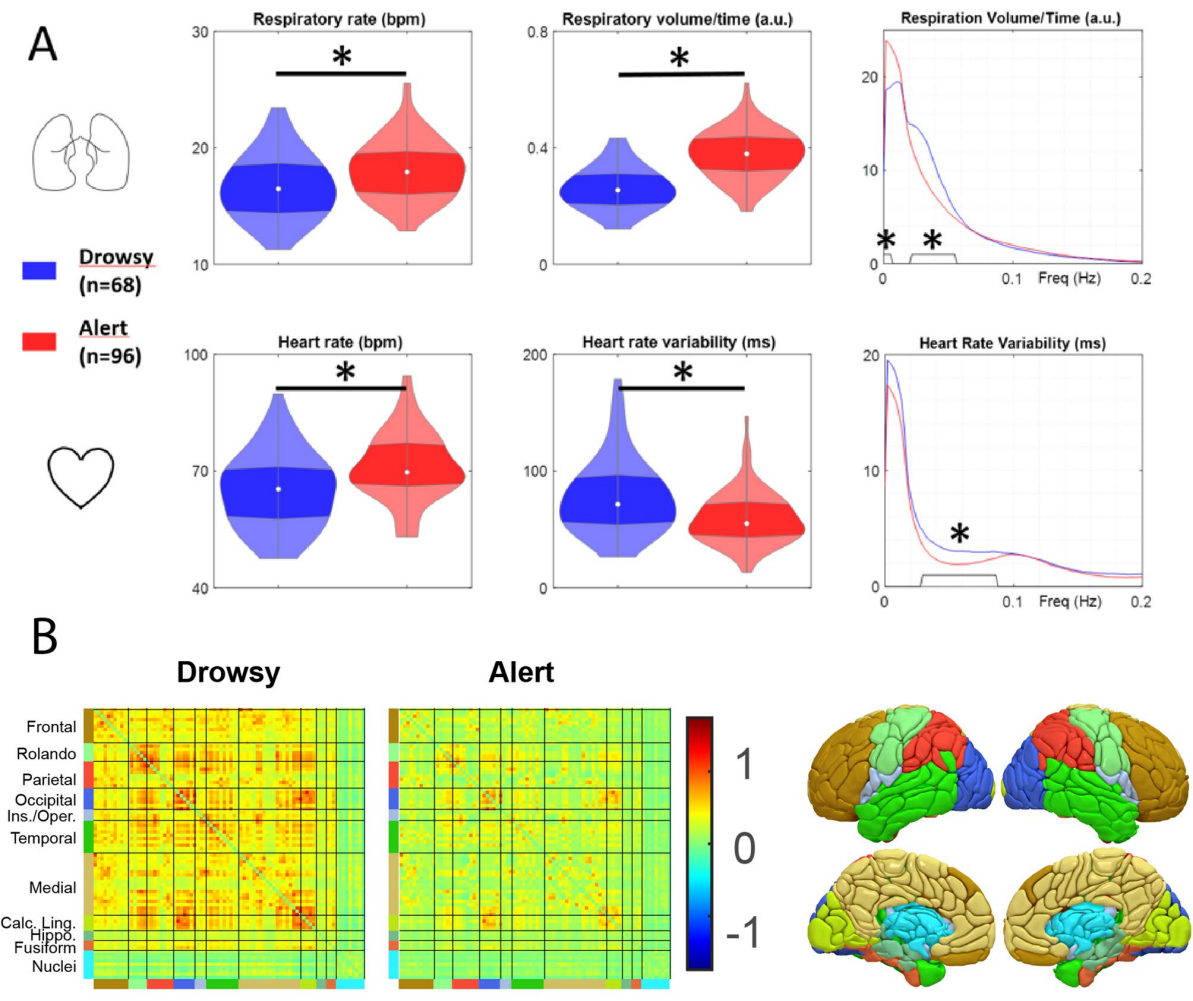
(**A**) Analysis of respiratory rate, volume per time, and frequency content (top) and heart rate, rate variability, and frequency content (bottom) for drowsy and alert subject groups (*: *p* < 0.05). (**B**) Group average FC matrices computed in the REF analysis. The color on the border of the matrices represents the areas shown in the 3-dimensional view of the atlas.

We conducted a group analysis to characterize the physiological variables of these two populations. Using the PhysIO toolbox^42^, we automatically detected each breath and heartbeat. Subsequently, all detected events were individually checked and manually corrected, if necessary, using custom software. The following variables were extracted: the time difference between two consecutive breaths on the respiratory movements signal to estimate the instantaneous breathing rate and the estimated Respiratory Volume per Time (RVT) (Fig. 1A bottom). Similarly, the time difference between two consecutive cardiac beats on the PPG signal was used to estimate instantaneous heart rate (i.e., the tachogram), and heart rate variability (Fig. 2B bottom) was assessed based on this tachogram. Finally, frequency analysis was performed on the RVT and tachogram using a multitaper spectrogram^43^.

### Resting-state processing

The preprocessing of RS-fMRI data is summarized below. For an in-depth explanation, refer to Tsuchida et al.^20^ and the supplementary materials. Initially, to ensure signal stabilization, the first 30 s of EPI data, consisting of 35 repetitions, were excluded. Afterwards, the remaining EPI volumes (1023) were subjected to spatial registration and corrected for geometric distortions. The RS-fMRI data were aligned with the T1-weighted images and non-linearly transformed into the Montreal Neurological Institute (MNI) stereotactic space using the normalization matrix from T1-weighted to MNI space. To compensate for residual movement effects (including translations, rotations, and their derivatives), as well as signals associated with fluctuations in white matter and cerebrospinal fluid and low-frequency variations below 0.01 Hz, regression-based amplitude correction was applied to each voxel's time series.

Two control quality markers, head motion (mHD: mean relative displacement^44^) and mean Framewise Displacement (mFD: mean of the instantaneous FD^45,46^), were derived from the movement parameters to quantify motion in two ways.

The global brain signal (GBS) was computed as the average of each brain voxel's BOLD signal time series. The signal variance of GBS (vGBS, standard deviation of the GBS values across time), which was previously reported to be linked to vigilance^27,47,48^, was also computed.

The FC between each pair of regions (384 regions based on the AICHA atlas^49^) was calculated as the Pearson correlation coefficient between the average time course of the voxels belonging to the respective regions. This resulted in the computation of 73,536 FCs in each 164 subjects. It should be noted that the AICHA atlas includes 344 cortical regions and 40 “deep nuclei” regions. Each nucleus is covered by a different number of regions (per hemisphere, 1 for the amygdala, 3 for pallidum/putamen, 7 for the caudate, and 9 for the thalamus). We will, after that, refer to deep nuclei as the subpart of the deep nuclei. *3D views of the atlas were computed using* Surf Ice 1.0.20190803, (https://www.nitrc.org/projects/surfice).

Finally, from the RS questionnaire, we extracted the question regarding awareness of sleepiness (QAS), which was answered on a four-level scale (“never, rarely, often, and all the time”).

### Correction of drowsiness effect

Four methods were tested to account for differences in FCs between subjects experiencing periods of drowsiness and those fully alert. The first two methods were applied to the time courses, while the last two were applied directly to the FC values. The first method involved constructing regressors of non-interest based on the two acquired physiological signals, using both RETROICOR^39^ and Physiological Response Function (PRF^38^). This method will be referred to as Ret-PRF. The PRFsc or M04 scan-specific model was used to estimate the cardiac and respiratory response functions. The correction level was evaluated by computing the correlation between the whole brain time courses and the estimated physiological response functions for both the heart and respiratory signals.

The second methodology is called Global Signal Regression (GSR, see review of Murphy et al.^40^). In this method, the GBS time series is regressed out from each voxel time series. Such methodology required estimating ~ 146 k parameters per subject (one per voxel of the gray matter).

The third method implemented a z-score normalization of each subject's FC distribution (Zs). The average (mFC) and standard deviation (sdFD) of the FCs (73,536 FCs) were computed independently for each subject. FC's values were z-scored by the classical operation: subtraction of the mFC and division by the sdFC.

In contrast, the fourth method implemented z-score normalization of the Gaussian of a mixture modeling of the FC distribution (Gaussian + two gamma functions), this method will be referred to as ZMix. Independently for each subject, the FC distribution was fitted by the sum of a Gaussian distribution (3 parameters, amplitude, mean, and standard deviations) and two gamma distributions (3 parameters per distribution amplitude, shape, and scale) one for the extremal positive correlation and one for the extremal negative correlation. To estimate the parameter, we used the implementation used in the melodic software (https://fsl.fmrib.ox.ac.uk/fsl/fslwiki/MELODIC). Following the estimation of the nine parameters, the FC’s values were z-scored by the classical operation, i.e., subtraction of the mean of the Gaussian distribution and division by the standard deviation of the Gaussian distribution.

### Statistical analysis

By implementing eight linear regression models, we investigated vigilance’s effect on global psychological and biological dependent variables. The vigilance (2 levels: drowsiness N = 68, alertness N = 96) was entered into the models as an independent predictor of 1/ the QAS extracted from the RS auto-questionnaire, 2/ the quality control markers for movement (mHD and mFD), 3/ the four physiological variables, and 4/ the vGBS. ANOVA component estimations and post hoc significant differences were reported at a *p*-value threshold 0.05 for all the variables. Similarly, we investigated the impact of vigilance variables on FC for each of the four correction methods by employing a linear regression model. We reported ANOVA component estimations and post hoc significant differences using a *p*-value threshold of 0.05, adjusted for multiple comparisons across 73,536 tests (calculated as 384*383/2) using the Bonferroni correction method. All analyses were controlled for age, sex, and total intracranial volume in the FC analysis.

The differences between the spectrograms (RVT and tachogram, see Physiological Signal Processing and Vigilance Assessment) were tested using Wilcoxon rank sum tests controlling the false discovery rate using the procedure described in Benjamini & Yekutieli^50^ that is guaranteed to be accurate for any test dependency structure.

### Synthesis of the FC differences across the five methodologies

We developed a methodology highlighting the regional co-evolution of the FC difference between the drowsy and awake state across the five methods (REF + 4 correction methods). The rationale was to cluster the regions with the same evolution across the five methods using a hierarchical decomposition algorithm (JMP software implementation, https://www.jmp.com). First, for each methodology, we built a square FC matrix of size 384 × 384 using the analysis results presented in the paragraph «Statistical analysis». These matrices represented the significance of the FC difference between two drowsy and alert states for each pair of regions of the AICHA atlas. We assigned a value of 1 if the FC was significantly higher (*p* < 0.05 Bonferroni corrected) in the drowsy state compared to the alert state, − 1 if it was significantly higher in the alert state compared to the drowsy state, and 0 otherwise. Secondly, we concatenated the five FC matrices and thus defined a 384 × 1920 matrice. We use the hierarchical decomposition with a ward cluster distance function to create the tree-like decomposition. The level of decomposition (4 clusters) was chosen to isolate clusters showing outliers of the distance to the parent node (see Supplementary Figure S1).

Lastly, we quantified the FC within and between each class and defined a metric called FC class (FCc). We calculated the FCcs as the difference between the number of significant FCs where drowsy was greater than alert and the number of significant FCs where alert was greater than drowsy. The total number of FCs normalized each FCc to scale its value between − 1 and 1.

## Results

### Vigilance effect on auto-evaluation and quality markers

On the self-report questionnaire, a higher frequency of sleepiness was consistently reported in the drowsy group compared to the alert group (t = − 5.03, df = 160, *p* < 0.0001). The two movement quality control markers used in the fMRI analysis were equivalent in both groups (mHD: F = 1.53, df = 160, *p* = 0.208 and mFD: F = 1.53, df = 160, *p* = 0.209).

### Vigilance effect on physiological variables

The results of the analysis of the physiological variables are summarized in Fig. 2A Regarding respiration, drowsy subjects exhibited a significantly lower respiratory frequency (t_Dr-Al_ = − 2.81, df = 160, *p* < 0.005) and Respiratory Volume per Time (RVT) (t_Dr-Al_ = − 8.6, df = 160, *p* < 0.0001) than the alert subjects. Frequency analysis of the RVT signal showed that the continuous component was more pronounced in the alert subjects. In contrast, the drowsy subjects showed more oscillations around 0.033 Hz, confirming the distinction between the two groups based on breathing pattern (oscillatory vs. stable breathing). In terms of heart rate, the drowsy subjects had a generally lower heart rate (t_Dr-Al_ = − 3.61, df = 160 *p* = 0.0004) and significantly higher cardiac variability (t_Dr-Al_ = 3.84, df = 160, *p* < 0.0002) than the alert subjects, particularly around the frequency of respiratory oscillations.

The correlation between the GBS and the PRF Respiratory-based correction was found to be higher in the drowsy group (z-corr_Dr_ = 0.54 ± 0.13) compared to the alert group (z-corr_Al_ = 0.35 ± 0.13) (t_Dr-Al_ = 8.04, df = 160, *p* < 0.0001). Conversely, the correlation for the PRF heart-based correction was higher in the alert group (z-corr_Al_ = 0.36 ± 0.14) compared to the drowsy group (z-corr_Dr_ = 0.28 ± 0.13) (t_Dr-Al_ = − 2.56, df = 160, *p* = 0.011).

### Vigilance Effect on FC of the Reference analysis (REF)

Group average FC matrices are shown in Fig. 2B. Cortico-cortical FCs are positive and higher in the drowsy group compared to the alert group. Between most of the deep nuclei, FCs are lower than cortico-cortical FCs and similar in both groups (bilateral Caudate-1–2–4–6–7, Pallidum-1, Thalamus-1–2–3–4–7–9). In addition, the drowsy subjects exhibit a higher global signal variance of GBS (vGBS) than the alert subjects (t_Dr-Al_ = 10.97, df = 160, *p* < 0.0001).

### Distributions of the FC after correction by the 4 methods

**Figure 3:**
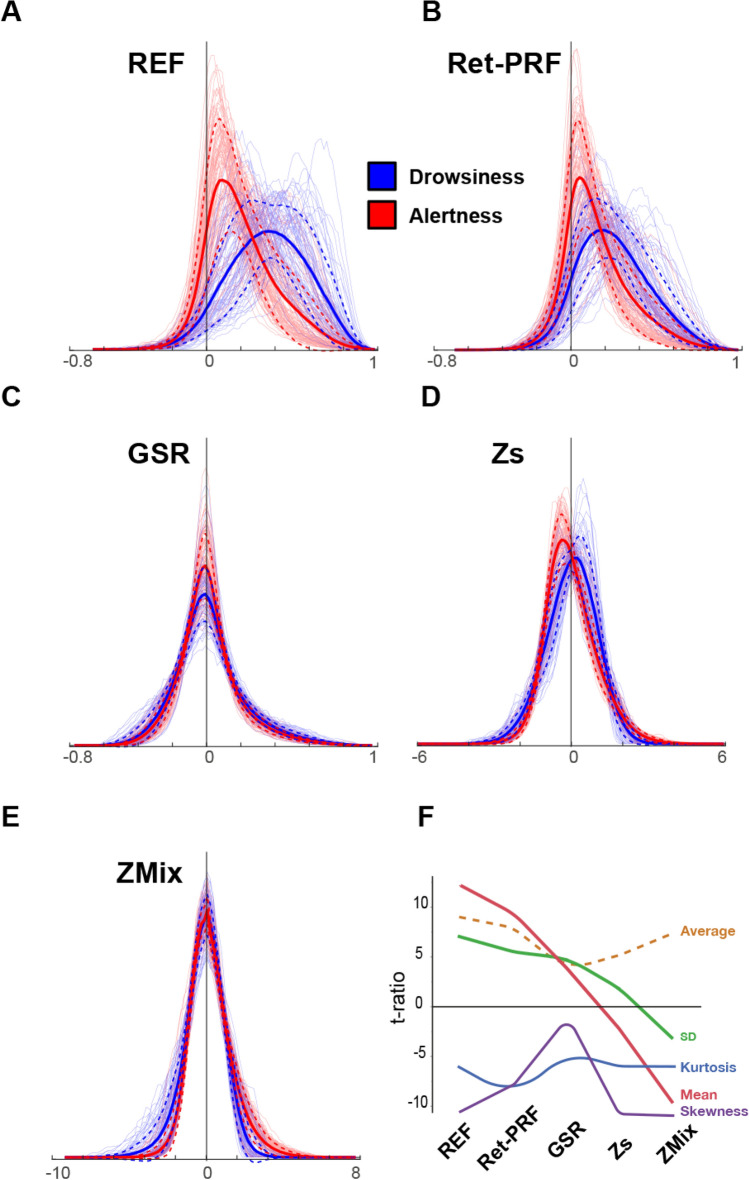
Histograms of inter-regional functional connectivity for the five analyses: (**A**) REF analysis, (**B**) Ret-PRF, (**C**) GSR (correction applied on the time course), and (**D**) Zs and (**E**) ZMix (correction applied on the FC values). The thick lines represent each class's average (Drowsy, Alert) subjects, and the dashed line represents the average plus or minus one standard deviation. The thin lines represent individual subjects. (**F**) T-ratio group differences of the first four moments of the individual histogram modeling. The dashed line represents the average of the absolute values of the four moments.

In the REF analysis, the drowsy subject’s histograms have high correlation values (Fig. 3A). The Ret-PRF technique (Fig. 3B) reduces the value and extent of both the REF distributions (drowsy and alert). The GSR technique (Fig. 3C) centers both distributions around null correlation values, while maintaining a slightly more pronounced spread of positive values in the drowsy group. The Zs technique (Fig. 3D) increases the spread of the alert group towards positive values, and the ZMix technique (Fig. 3E) enhances this effect. The quantification of the group differences (t-ratio) based on the first four moments (mean, standard deviation, skewness, and kurtosis) of the individual histogram distributions is shown in Fig. 3F. The average absolute t-ratio of the four moments is minimal for the GSR correction (see dashed curve in Fig. 3F).

### Comparison of 4 methods for correcting the effects of vigilance on FC

**Figure 4:**
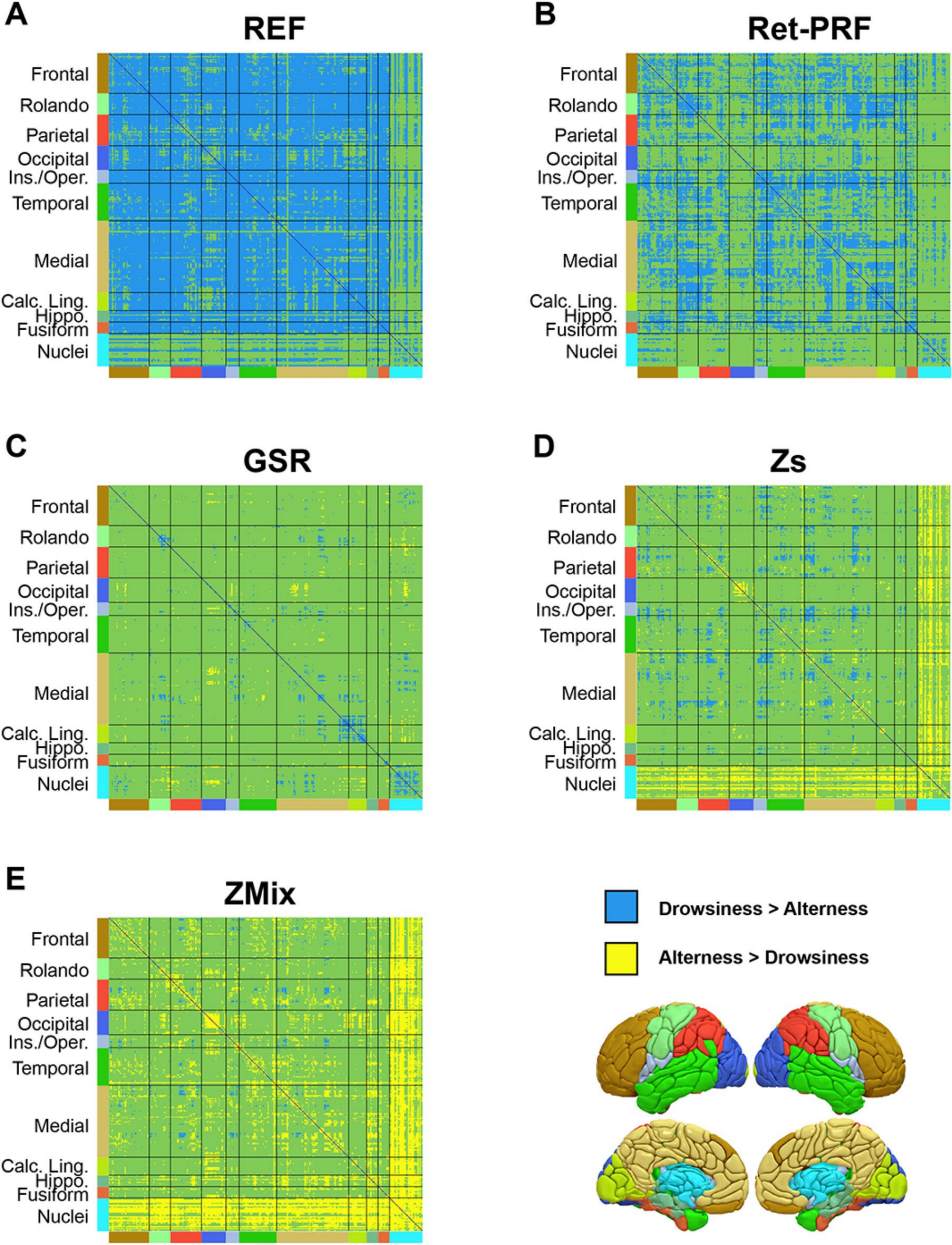
Significant effect of vigilance on inter-regional FC calculated using the AICHA atlas (384 regions) for different analysis approaches: (**A**) Reference analysis (REF), (**B**) Ret-PRF correction, (**C**) GSR correction, (**D**) Zs correction, and (**E**) ZMix correction. The green represents region pairs with no significant difference, while blue and yellow indicate significant differences (*p* < 0.05 Bonferroni corrected). The color bordering the matrices corresponds to the areas depicted in the lower-right three-dimensional view of the atlas.

Figure 4 and Table 1 show the quantitative analysis of significant differences between drowsy and alert subjects in the REF analysis and the four analysis methods. In the REF analysis (Fig. 4A), 65% of the FCs were higher in drowsy subjects than in alert subjects, while none showed the opposite pattern. This percentage decreased to 31% with the Ret-PRF analysis (Fig. 4B) and fell below 5% with the other three techniques. Conversely, the number of alert over drowsy FCs remained zero with the Ret-PRF analysis but increased from 1% with GSR (Fig. 4C) to 6% with Zs (Fig. 4D) and 18% with the ZMix analysis (Fig. 4E). The GSR analysis had the lowest number of significant FCs at 3.3%.

**Table 1:** Percentage of the significant (Bonferroni corrected) vigilance effect on the inter-regional FC.

Type of correction	Alert > drowsy (%)	Drowsy > alert (%)	Sign. FCs (%)
REF	0	65.9	65.9
Ret-PRF	0	30.7	30.7
GSR	1.0	2.3	3.3
Zs	6.2	4.4	10.6
ZMix	18.3	0.8	19.2

**Figure 5:**
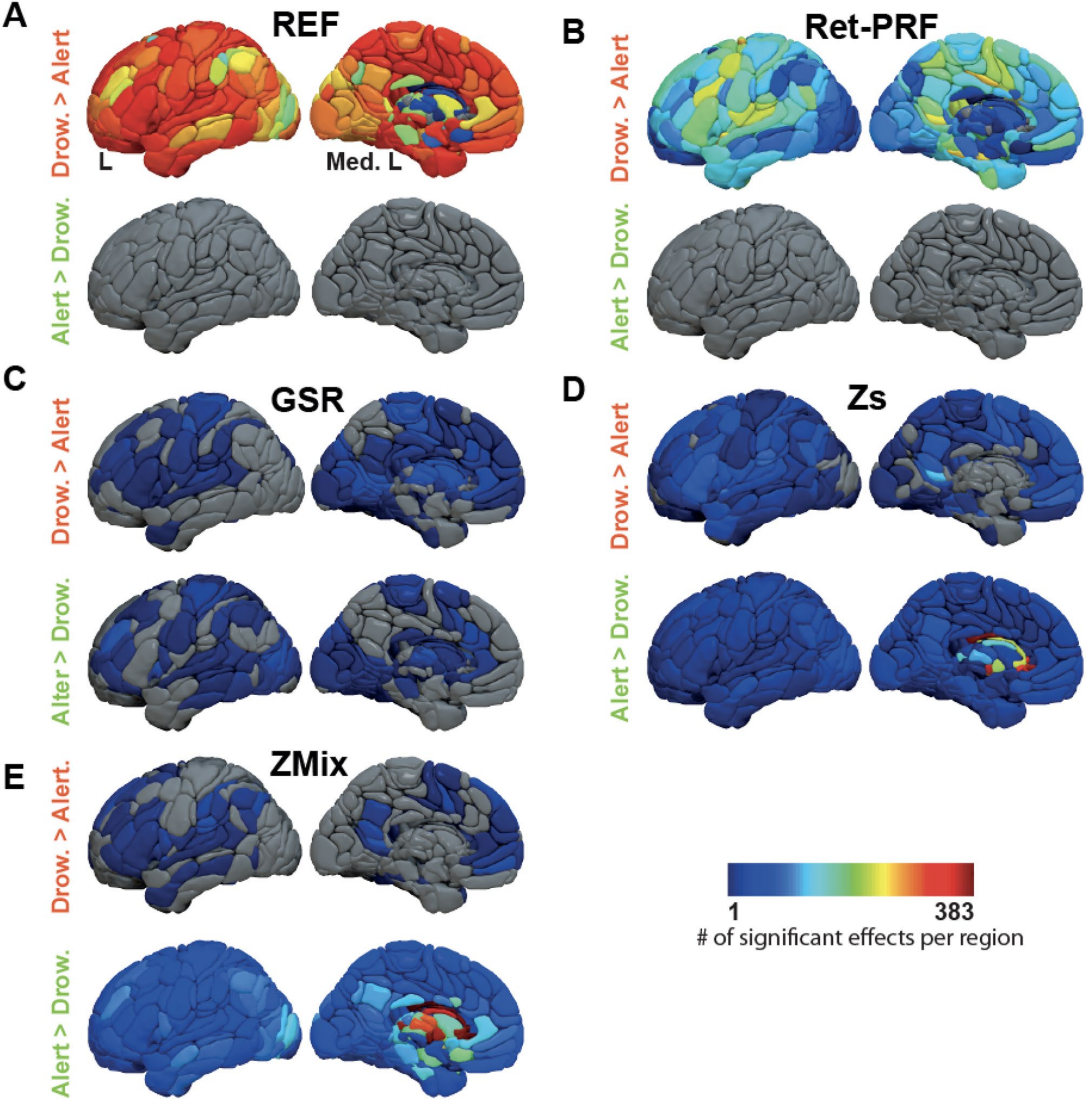
Synthesis of the vigilance effect on inter-regional FC per region (only the left hemisphere is presented) and analysis approaches: (**A**) REF analysis, (**B**) Ret-PRF correction, (**C**) GSR correction, (**D**) Zs correction, and (**E**) ZMix correction. In each segment of the figure, the upper and lower lines represent the number of significant effects for drowsiness and alertness, respectively, across regions.

The regional specificity of these differences for the left hemisphere is illustrated in Fig. 5. For each analysis and each region of the atlas, the numbers (drowsy over alert and alert over drowsy) of significant FCs, including this region, were computed. Two maps were created for each analysis: the regional specificity of the drowsy over alert difference and the reverse.

In the REF analysis (Fig. 5A upper line), only drowsy over alert FCs were observed in all cortical regions, with higher magnitudes in the frontal areas and the occipital lobes. Conversely, most deep nuclei (bilateral Caudate-1–2–4–6–7, Pallidum-1, Thalamus-1–2–3–4–7–9) showed no significant differences (Fig. 5A upper line).

The Ret-PRF analysis (Fig. 5B upper line) reduced the drowsy over alert values in all cortical regions, while the GSR analysis (Fig. 5C upper line) further reduced it.

With an alert over drowsy low magnitude in the GSR analysis (Fig. 5C lower line), the magnitude increases in the Zs (Fig. 5D lower line) and further in ZMix (Fig. 5E lower line). In this latter analysis, the deep nucleus captured the specificity of the alert state. Among these deep nuclei, those found to be non-significant in the REF analysis get a significant alert over drowsy FCs with the majority of the atlas regions.

**Figure 6:**
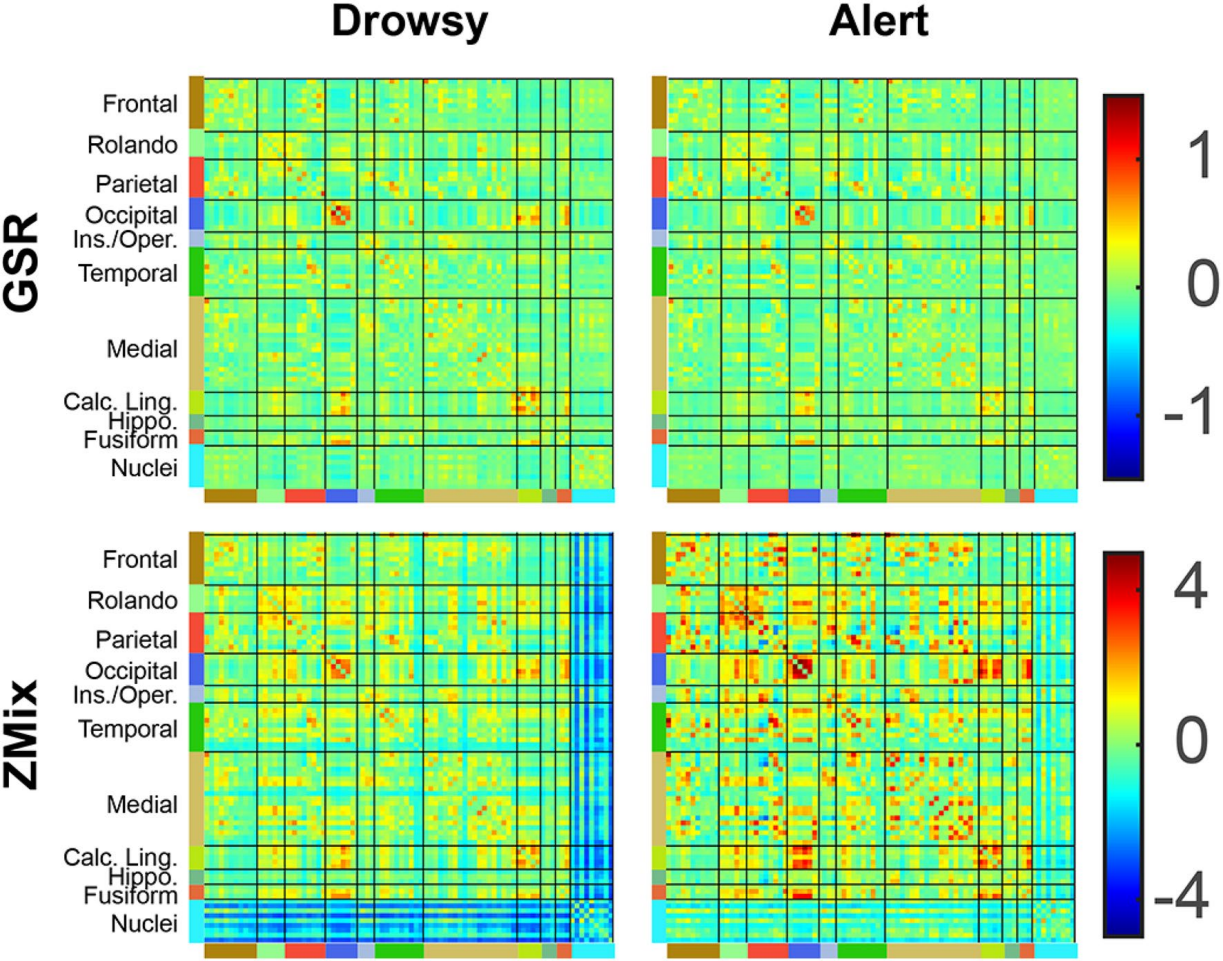
Average z-score FC computed for drowsy and alert subjects using GSR and ZMix corrections.

Figure 6 shows the average FC matrices for drowsy and alert subjects in the GSR and ZMix analysis. As expected, the GSR-derived matrices were similar, while the alert matrix from the ZMix analysis showed larger values than the drowsy matrices.

### Synthesis of the vigilance induced FC differences across the five methodologies

**Figure 7:**
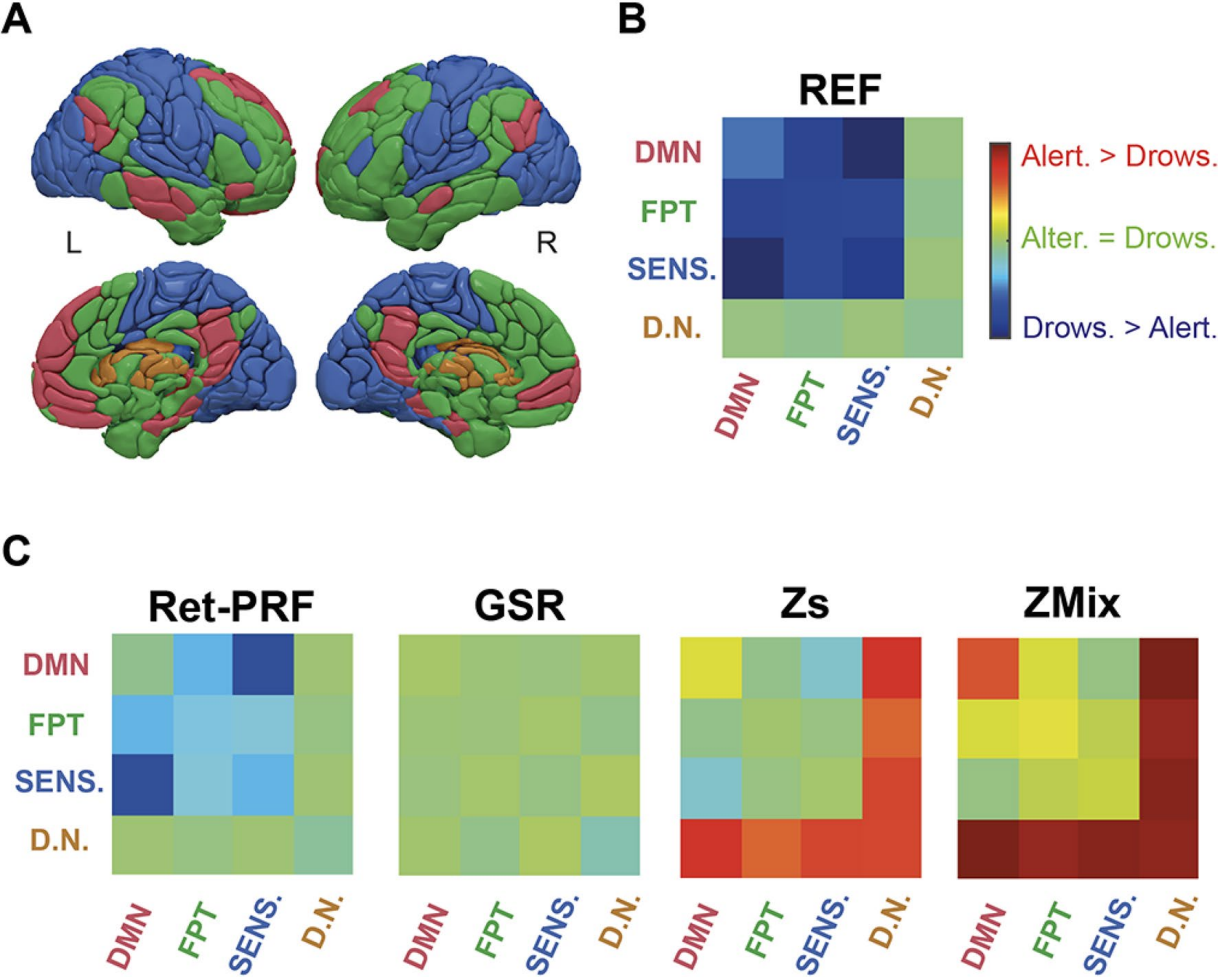
(**A**) Hierarchical decomposition-derived parcellation into four classes : Default mode network/red (DMN), Fronto-Parieto-Temporal/green (FPT), Sensory related/blue (SENS.), Deep nuclei/brown (DN) (**B**) Significant vigilance effect on the intra- and inter-class FC (FCcs) for the reference analysis (REF). (**C**) Significant vigilance effect FCcs for the four types of corrections: Ret-PRF, GSR, Zs, and ZMix.

Figure 7 provides a summary of the FC differences observed between drowsy and alert subjects. To combine brain regions of interest, we chose a four-class partition, roughly corresponding to the cortical divisions into sensory, executive, default mode region^51^, and deep nuclei classes (Fig. 7A). This partition is similar to the gradient-based decomposition proposed by Margulies et al.^52^.

In the REF analysis (Fig. 7B), FC is higher in drowsy than in alert subjects with respect to cortico-cortical relations, whereas there is no difference in FC between the 2 groups in the cortico-nuclear relations.

All four correction methods (Fig. 7C) reduce the difference in FC between drowsy and alert subjects. The Ret-PRF and GSR methods are effective on cortico-cortical relations without altering cortico-nuclear relations, with the GSR method being more effective than Ret-PRF.

The Zs and ZMix methods are also more effective than Ret-PRF in attenuating the two groups cortico-cortical differences, but on the other hand they induce cortico-nuclear differences that were not present in the REF analysis, with the FC of alert subjects becoming higher than that of drowsy subjects.

The DMN regions that showed the least drowsy FC superiority in the REF cortico-cortical relations show stronger FC in alerts when corrected with ZMix, following the same trends as the deep nuclei.

## Discussion

The study aimed to investigate various methods for modulating the effects of drowsiness on FC, as estimated from a RS-fMRI dataset. Our analysis focused on identifying a group primarily consisting of drowsy participants and another primarily consisting of awake participants, upon which we assessed group differences in FCs.

We will first briefly discuss the data preprocessing that was the common ground of the reference and the four correction methods. We will then discuss the physiological markers associated with and used for the two groups’ construction. Finally, we will discuss the results of each of the five methodologies in relation to the literature and then the limitations.

Various technologies have been developed to mitigate sources of variance associated with movement and susceptibility effects, as summarized in the review by Caballero-Gaudes et al.^19^. These technologies aim to reduce noise and enhance the quality of fMRI data through approaches such as spatial smoothing or region region of interest analysis. The basic process also involves temporal registration and stereotaxic normalization of fMRI data to a surface or volume atlas. However, temporal slice registration was not applicable in our dataset due to a short repetition time and multiband acquisition, rendering the correction meaningless. Our focus on deep nuclei behavior also led us to choose volumetric stereotaxic normalization. These preprocessing steps led to the “reference” (REF) analysis, from which we deliberately excluded the physiological signal-based corrections that were explicitly studied (see below for discussion).

Our study was motivated by analyzing the RS auto-questionnaire completed by all subjects in the MRi-Share cohort^22^. This questionnaire included questions directly related to vigilance, such as falling asleep during the acquisition, and indirectly related questions about the number of hours of sleep preceding the acquisition. The responses indicated that 50% of the participants reported some drowsiness, suggesting that vigilance was an issue in this dataset. However, we refrained from using these indexes to classify the two groups, as drowsiness is not always accurately remembered^53^, and some subjects may choose not to report behaviors that contradict the instructions. The classification of sleep stages is typically based on polysomnography divided into 30-s windows (AASM). Still, this approach may be less suitable for assessing rapid alternations between wakefulness and light sleep observed during drowsiness^54–56^, especially in an fMRI examination^21^. Instead, we identified drowsiness using a specific physiological marker: oscillating breathing, observed when a subject falls asleep, and that disappears quickly as sleep becomes well-established^41^. Note that the vGBS, an indirect BOLD-derived marker of the vigilance^48^ was significantly higher in the drowsy compared to the alert group.

In our study, the mean frequency analysis revealed differences between the drowsy and awake populations in oscillations with a period of approximately 30 s, consistent with the characteristics of oscillatory breathing observed at sea level^36^. Furthermore, the respiratory rate, heart rate, and estimated respiratory volume per minute were significantly lower in the population exhibiting oscillatory breathing. In contrast, the cardiac variability of this same population was higher. These observations align with the physiological variations associated with sleep^57^, confirming that this population experiences a higher level of drowsiness than those without oscillating breathing.

The reference analysis yielded two main findings. First, drowsy subjects generally exhibited higher FC values than alert subjects. As shown in Fig. 3a, this increase varied across drowsy individuals, resulting in an enlarged average histogram for the drowsy group. This variability may reflect different patterns and durations of drowsiness experienced during the 15 min of the experiment. Second, no FC wakeful above drowsy FC group difference was significant, whereas 70% of the drowsy above wakeful differences were significant. These results align with previous findings by Tagliazucchi^21^ regarding the difference between N1 and wake, as well as the observed differences in FC between sleepy and alert conditions (Fig. 2 of Wang et al.^58^) and wavelet-based analysis of cortico-cortical connections^59^. They are also consistent with the overall increase in network connectedness observed by Larson-Prior et al.^60^. Nguyen et al.^61^, using functional near-infrared imaging, also reported a significant increase in correlation during N1 compared to wake, with N2 being more similar to wake than N1, which contrasts with the findings of Tagliazucchi et al.^21^.

By applying the state-of-the-art physiological-based correction method, Ret-PRF^38,39^, the differences between the two states decreased but remained significant in all cortical regions. This technology corrects the noise created by the heart and respiration^39^ and the hemodynamic responses to locally occurring neural processes^38^. However, this correction method does not capture local spatial specificities accurately, which is a common challenge in task-based fMRI analyses, where the hemodynamic response function is often assumed to be a fixed function (e.g., SPM hemodynamic response function). Nonetheless, the level of correction provided by the respiratory-derived regressor was significantly greater in the drowsy group than in the alert group, confirming that the drowsy state involves neural-based modifications of the respiratory function reflected in the fMRI signal.

Global regression-based correction proved the most effective among the three correction methodologies tested. Quantitatively only 3.3% of the independent components (FCs) remained significantly different between the two states. Contrary to the controversy, Murthy et al.^40^ concluded that using such correction methods is not inherently “right or wrong” but depends on the scientific question. If the goal is not to study sleep stages, drowsiness, a common confound in RS studies^21^, should be accounted for. Global regression-based correction is currently the most effective approach for reducing variability in FC associated with the drowsy state and making the data of drowsy subjects comparable to data from alert subjects. In hypervigilance, Wong et al.^48^ demonstrated that the amplitude of the GBS low-frequency oscillation was lower with caffeine than without. We thus can postulate that the whole spectrum of the variation of vigilance from drowsiness to hypervigilance will be corrected using the global regression-based method. However, it's crucial to acknowledge that this correction method could be considered overly aggressive since it treats each voxel time series independently. As a result, certain significant features might be removed from the data through such a correction process. An example of this effect is evident in the language lateralization study conducted by McAvoy et al.^62^. In conclusion, the most quantitatively effective correction might not always be the most appropriate choice.

The other two correction methodologies were designed based on the observation that drowsiness impacts the entire distribution of FC. Therefore, a global correction approach could be applied. In the case of the pseudo-Gaussian distribution, the most parsimonious correction method is the z-score-based transformation (Zs). This correction equalizes the individual distributions but also shifts the maximum values of the alert subject distribution toward negative values. On the other hand, the mixture model-based correction (ZMix) employs a more complex modeling approach that perfectly aligns the drowsy and alert distributions in both value and amplitude. With the ZMix correction, most differences between the two states manifest as greater FCs in the alert state, with 70% of those including a deep nucleus. For the thalamus (included in 27% of the FCs), several studies have reported a loss of FCs with cortical regions during early NREM sleep (reviewed in Tagliazucchi et al.^63^). The significant predominance of the FCs intra-DMN in the alert state aligns with the expected role of the DMN compared to other executive or sensorial networks at rest. Historically, the DMN was characterized as maintaining activity during conscious RS, contrasting with task-related activities, as described by Mazoyer et al.^2^. After two decades of research, there is now consistent evidence that the DMN supports mind wandering and the occurrence of spontaneous, independent thoughts, as reviewed by Menon^51^. Moreover, its role is consistent with the decoupling observed during NREM sleep^26,60,64–66^.

One of the striking messages of this study is that depending on the processing methods used (Ret-PRF, GSR, Zs, or ZMix), the effects of drowsiness on FCs can drastically change or even be reversed without knowing which is the best technique. We tentatively interpret our results in light of classical sleep electrophysiology data, hoping it will inspire further research to address the limitations of our current study (refer to the following paragraph for details).

Studies based on electrophysiological data clearly show that falling asleep is progressively accompanied by a relaxation of the muscles and sensory isolation of the brain to allow the descent into the deeper stages (see for review Ogilvie et al.^54^). These functions are under the control of cortico-subcortical loops controlling sensory input via the thalamus and motor control via the basal ganglia. From a connectivity point of view, the progressive isolation of the cortex from the world should decrease FC between the deep brain nuclei and the cortex in sleepy subjects. This picture is observed with the Zs and ZMix corrections (Fig. 7C).

Moreover, drowsy subjects monitored during an RS-fMRI examination can alternate rapidly between wake and sleep states, contrary to those monitored in classical sleep studies where drowsiness gives rapidly way to light then deep NREM sleep. This alternation can induce notable hemodynamic variations. Indeed, the descent into sleep is accompanied by physiological phenomena such as a decrease in heart and respiratory rate or an increase in CO_2_ partial pressure^57^. In contrast, micro-arousals, especially during napping, are accompanied by a sympathetic discharge causing tachycardia and peripheral vasoconstriction, among other things^67^. Thus, subjects who doze off in MRI show cardiorespiratory variations that can alter the MRI signal^32,68^. Finally, these global and periodic variations in phase with rapid sleep–wake alternations can simulate cortico-cortical pseudo connectivity.

To summarize, the differences in FC observed between drowsy and alert subjects, according to the correction techniques used, reflect different mechanisms. On the one hand, the differences in FC, mainly cortico-cortical, observed between the two populations in the REF analysis, which persist after the Ret-PRF correction and disappear after the GSR correction, are primarily a result of the iterative cardiorespiratory variations induced by the vigilance fluctuations. On the other hand, the differences in connectivity concerning cortico-subcortical relationships that appear after the Zs and ZMix corrections reflect the progressive isolation of the cortex from the deep structures observed during the descent into sleep.

## Limitations

The primary limitation of this study stems from the absence of an EEG-based marker for classifying sleep stages. This was due to the challenges of simultaneously acquiring EEG signals during fMRI scans. Incorporating EEG measurements would have prolonged the acquisition time beyond what was deemed acceptable for student participants. However, while the standard classification^69^ is particularly well suited to the study of sleep, it is less effective at capturing the dynamics of sleep onset^55,56^, especially if subjects are instructed not to fall asleep^30,53^. Oscillatory respiration has proven to be a valuable marker for drowsiness, but it is not guaranteed to be observed in all subjects, particularly in older individuals. Additionally, specific parameters of the scanner's physiological signal acquisition system, such as the gain, could not be adjusted, resulting in the exclusion of 16% of the subjects. Consequently, due to the lack of sleep stage characterization, the processing methods employed in this study may have underestimated the number of drowsy subjects. There is potential for improvement in this regard, particularly if it becomes feasible to accurately identify « light » sleep periods, which comprise most of the drowsiness period.

## Conclusion

In conclusion, our study demonstrates that the alternation between drowsy and alert phases during the classical RS paradigm significantly influences the variability of FC. A global regression is a practical approach for reducing this variability among the different processing methodologies. However, the Gaussian-based normalization technique yields the most accurate fit when comparing the FC between drowsy and alert subjects to the traditional sleep neurophysiological framework.

### Supplementary Information

Supplementary Information.

## Data Availability

Due to French regulations regarding the sharing of medical imaging data, individual raw data used for this study cannot be shared through a public repository. Instead, to access i-Share and MRi-Share de-identified data, a request can be submitted to the i-Share Scientific Collaborations Coordinator (ilaria.montagni@u-bordeaux.fr) with a letter of intent (explaining the rationale and objectives of the research proposal), and a brief summary of the planned means and options for funding.
