# Supplementary material for: Modulate the impact of the drowsiness on the resting state functional connectivity
Source: Sci Rep. 2024 Apr 15;14:8652. doi: 10.1038/s41598-024-59476-8 (PMC11018752; doi:10.1038/s41598-024-59476-8)
Supplement: Supplementary file 1 — Supplementary Information. [file 41598_2024_59476_MOESM1_ESM.docx]

**Resting-state processing**

The preprocessing of RS-fMRI data followed state-of-the-art standards outlined in Tsuchida et al. 2021 {Tsuchida, 2021 #6072}. First, using T1-weighted acquisition, normalization to the MNI template and segmentation into three tissue classes (GM: Gray Matter, WM: White Matter, CSF: Cerebrospinal Fluid) was performed. To ensure signal stabilization and eliminate subject responses to experimental onset, such as scanner noise, the initial 29.75 seconds of EPI data were removed (35 repetitions). The remaining EPI volumes (1023) underwent spatial registration, and B0 acquisitions were utilized to compute a B0 field map, which was then employed to correct for geometrical distortions in the EPI images. The average registered and corrected EPI data were further registered to the T1-weighted image, and each EPI volume was interpolated into the MNI stereotactic space. The time courses of the six parameters describing movement across time and the average BOLD signal in the eroded WM and CSF were extracted. Note that the WM and CSF masks were eroded 3 and 2 times, respectively, to ensure that no partial volume effect will make BOLD signal of gray matter included in the average BOLD signal of the two tissues. Erosion is a mathematical morphological operation that iteratively reduces the size of a binary-defined region by removing an outer band of 1 voxel (1mm in our case) of thickness at each iteration. A set of nuisance variables was constructed using the six movement parameters and their derivatives, the WM and CSF BOLD time courses, and sine and cosine time series corresponding to temporal filtering frequencies below 0.01 Hz. Subsequently, temporal filtering and nuisance regression were performed in a single regression model to minimize potential errors in data denoising {Caballero-Gaudes, 2017 #6138} using AFNI 3dREMLfit. This analysis was referred to as the Reference analysis (REF) after that.

**Synthesis of the FC differences across the five methodologies**

*
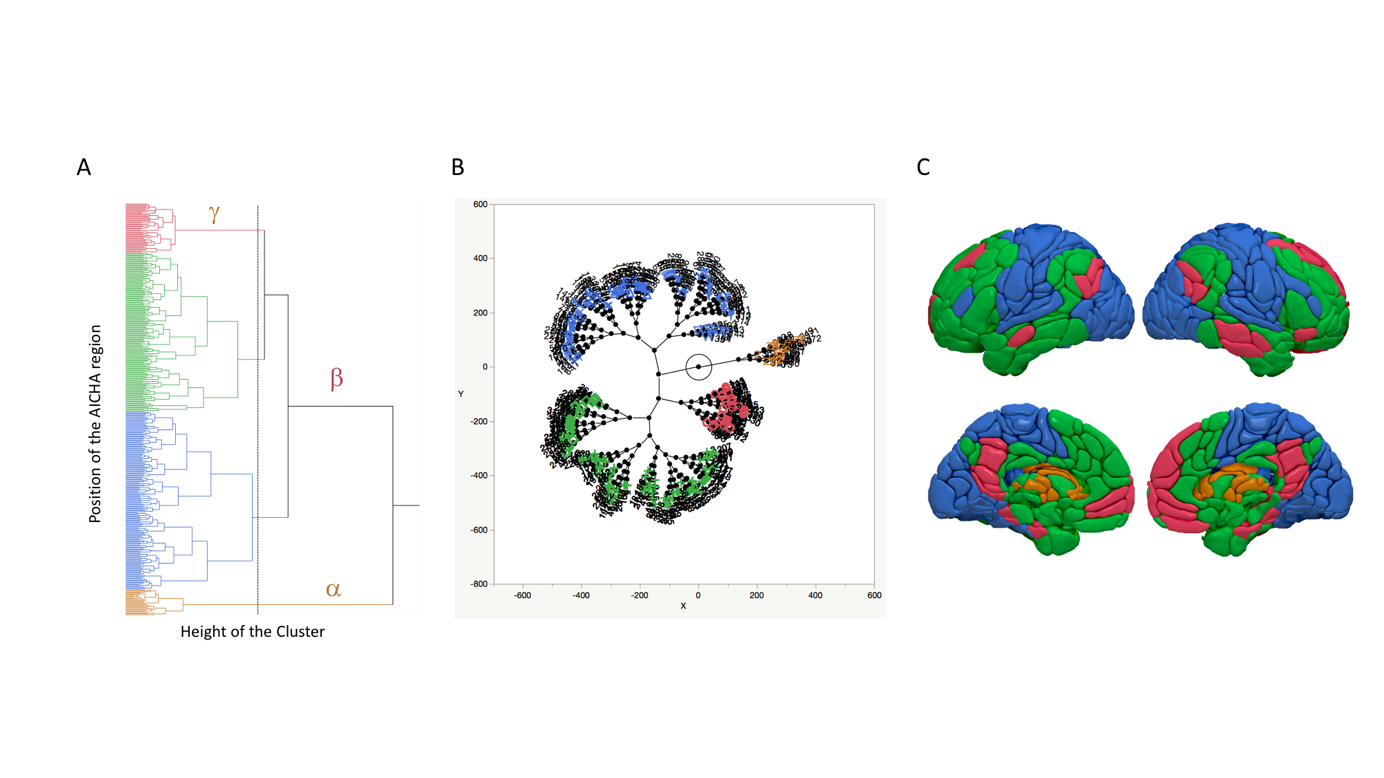
*

*Supplementary Figure 1: A) Tree of the hierarchical decomposition of the concatenation of the five matrices shown in Figure 4. α, β, γ are the edges outliers of the node-to-node distance distribution. The vertical line materializes the chosen level of decomposition in 4 clusters (colored red/green/blue/brown). B) Constellation plot of the hierarchical decomposition (JMP software implementation, https://www.jmp.com). C) Mapping of the clustering on the 3D AICHA atlas rendering.*
